# Primary Cerebral Lymphoma With Isolated Vitreoretinal and Cerebral Recurrences Without Meningeosis: A Case Report

**DOI:** 10.7759/cureus.75432

**Published:** 2024-12-09

**Authors:** Josef Finsterer, João Gama Marques

**Affiliations:** 1 Neurology, Neurology and Neurophysiology Center, Vienna, AUT; 2 Treatment Resistant Schizophrenia Outpatient Clinic, Júlio de Matos Hospital, São José Local Health Unit, Clinical Academic Center of Lisbon, Lisbon, PRT; 3 University Clinic of Psychiatry and Medical Psychology, Lisbon School of Medicine, University of Lisbon, Lisbon Academic Medical Center, Lisbon, PRT

**Keywords:** brain, lymphoma, polyneuropathy, vitreoretinal involvement, vitritis

## Abstract

Primary central nervous system lymphoma (PCNSL) is a diffuse, large B-cell lymphoma affecting the brain, spinal cord, leptomeninges, or eyes. A patient with a recurrence of a previous PCNSL manifesting as an isolated vitreoretinal disease without central nervous system (CNS) involvement and a second cerebral recurrence without vitreoretinal involvement has not yet been reported. The patient is an 86-year-old man with PCNSL of the left cerebellum diagnosed at the age of 82 years and treated with suboccipital trepanation and resection of the lesion followed by chemotherapy. At the age of 84, he suffered a first recurrence, which manifested as vitreoretinal lymphoma on the right and later also on the left eye, without CNS involvement. The recurrence was treated by vitrectomy on both sides. At the age of 86, the patient suffered a second recurrence, which manifested as infratentorial lesions treated by radiotherapy. He was discharged with dexamethasone, entecavir, valacyclovir, amphotericin-B, candesartan, tramadol, trazodone, and pantoprazole. This case demonstrates that PCNSL can recur with isolated secondary ophthalmologic lymphoma and a second time with isolated cerebral lymphoma. Although glucocorticoids, chemotherapy, and radiotherapy can be highly effective, the long-term outcomes of patients with PCNSL are not as favorable.

## Introduction

Primary central nervous system (CNS) lymphoma (PCNSL) is a diffuse, large B-cell lymphoma that exclusively affects the brain, spinal cord, leptomeninges, or eyes [1]. The pathophysiology of PCNSL is incompletely understood, but there is evidence that immunoglobulins block CNS proteins, that signaling via the B-cell receptor, toll-like receptor, or NF-κB is impaired due to mutations in genes involved in these pathways, or that T cells, macrophages, microglia, endothelial cells, cytokines, and chemokines are dysfunctional [1]. The transformation of B cells probably begins outside the CNS before the cells migrate into the CNS [2]. Immunosuppression is a risk factor for the development of PCNSL.

In some cases, PCNSL can be complicated by ocular involvement due to optic nerve spread or hematogenous dissemination, manifesting as optic neuritis, retinitis, retinal vasculitis, uveitis, vitritis, leopard spot appearance, Bruch's membrane/RPE infiltrations or ellipsoid zone disruption [3,4]. In a retrospective study of 46 patients with PCNSL, ocular involvement was found in 28% of cases [5]. Vitritis is the most common ocular manifestation of PCNSL [6]. Vitreoretinal involvement can be primary or rarely secondary [7]. Primary and secondary ocular lymphomas are often difficult to diagnose and are frequently confused with infectious ocular diseases [7]. A variable combination of MYD88-L265P mutation in the aqueous humor or vitreous and positive cytology/histology allows a definitive diagnosis [4]. Treatment of PCNSL with ocular involvement is based on glucocorticoids and high-dose methotrexate (MTX) chemotherapy as the cornerstone of first-line polychemotherapy [8]. After completion of MTX-based treatment, a consolidation strategy is often required to prolong the duration of the response. This consists of radiation, maintenance therapy, non-myeloablative chemotherapy, or myeloablative treatment, possibly followed by autologous stem cell transplantation (ASCT) [8]. Despite correct diagnosis, the outcomes of patients with vitreoretinal involvement of PCNSL are often not good [9]. However, there are no guidelines for diagnosing and treating vitreoretinal B-cell lymphomas [3]. To our knowledge, a patient with a relapse of a previous PCNSL manifesting as an isolated vitreoretinal disease without CNS involvement and a second relapse in the brain without vitreoretinal involvement has not been reported.

## Case presentation

**Figure 1:**
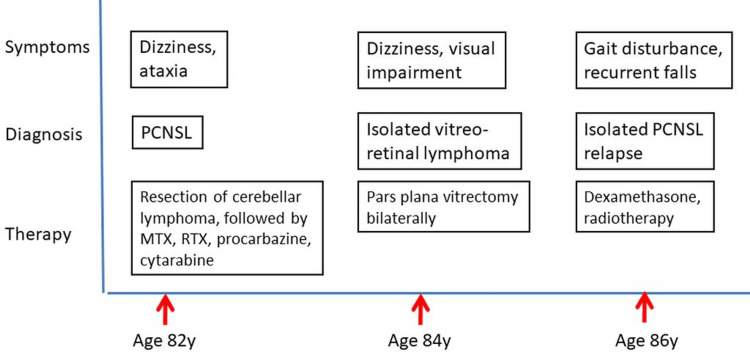
Disease course since the first diagnosis of PCNSL at the age of 82. PCNSL: Primary central nervous system lymphoma

The patient is an 86-year-old man with PCNSL, which was first diagnosed at the age of 82. At that time, he presented with dizziness and ataxia due to lesions in the left cerebellar hemisphere. He then underwent trephination and resection of these left cerebellar lesions, which were histologically diagnosed as primary diffuse large B-cell CNS lymphoma with CD20+, CD45+, Ki67% (60%), Mum1 20%-30%, and CD138 lymphocytes. Subsequently, the patient was treated with MTX, which was replaced by rituximab (RTX), MTX, and procarbazine (RMP). Procarbazine later had to be discontinued due to liver toxicity. Between November 2020 and February 2021, the patient received nine cycles of MTX, cytarabine, and RTX (MATRIX) (Figure 1). His medical history also included arterial hypertension, spondylodiscitis L2/3, spontaneous fracture of vertebra L1, osteoporosis, hepatitis B, and cytomegalovirus infection.

At the age of 84, he developed dizziness and progressive visual impairment. Cerebral MRI showed no changes compared to a previous examination in 2020 and PET-CT showed no structure with hypermetabolism. The ophthalmologists diagnosed extensive vitritis on the right and suspected vitreoretinal lymphoma, so a right pars plana vitrectomy and after vitritis in the left eye, a left pars plana vitrectomy were performed. Vitreal cytology revealed the variant MYD88 c.794T>C p.(Leu265Pro), suggesting a B-cell lymphoma, with no other evidence of lymphoma. The CSF examination did not reveal meningeosis.

**Figure 2:**
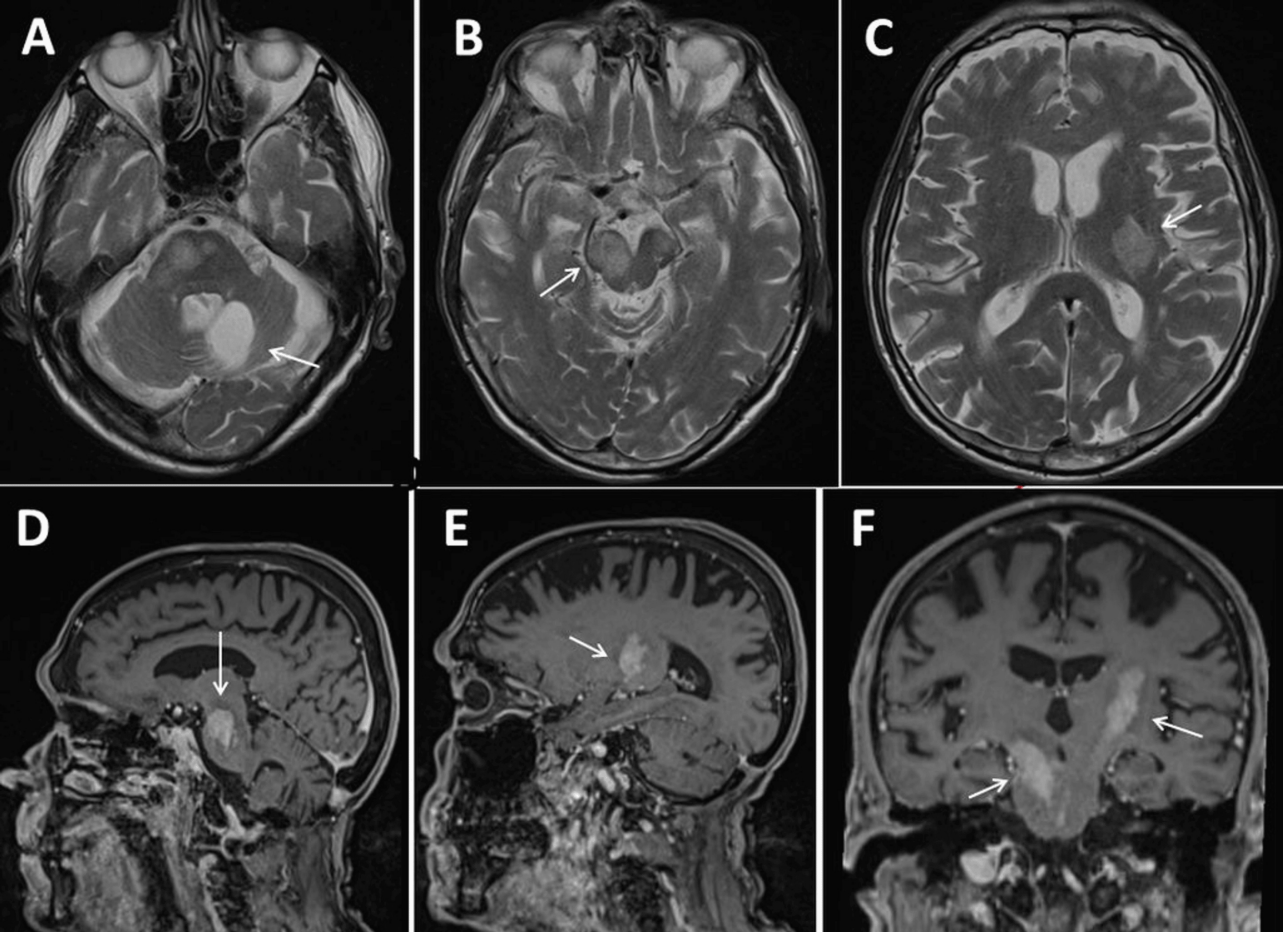
Cerebral MRI at 86 years of age showing the old surgical defect in the left cerebellum after resection of the lymphoma (A). Cerebral recurrence of PCNSL in the predunculi cerebri (B), the left basal ganglia (C) on axial images, on sagittal images (D, E), and on coronary images (F). PCNSL: Primary central nervous system lymphoma

**Figure 3:**
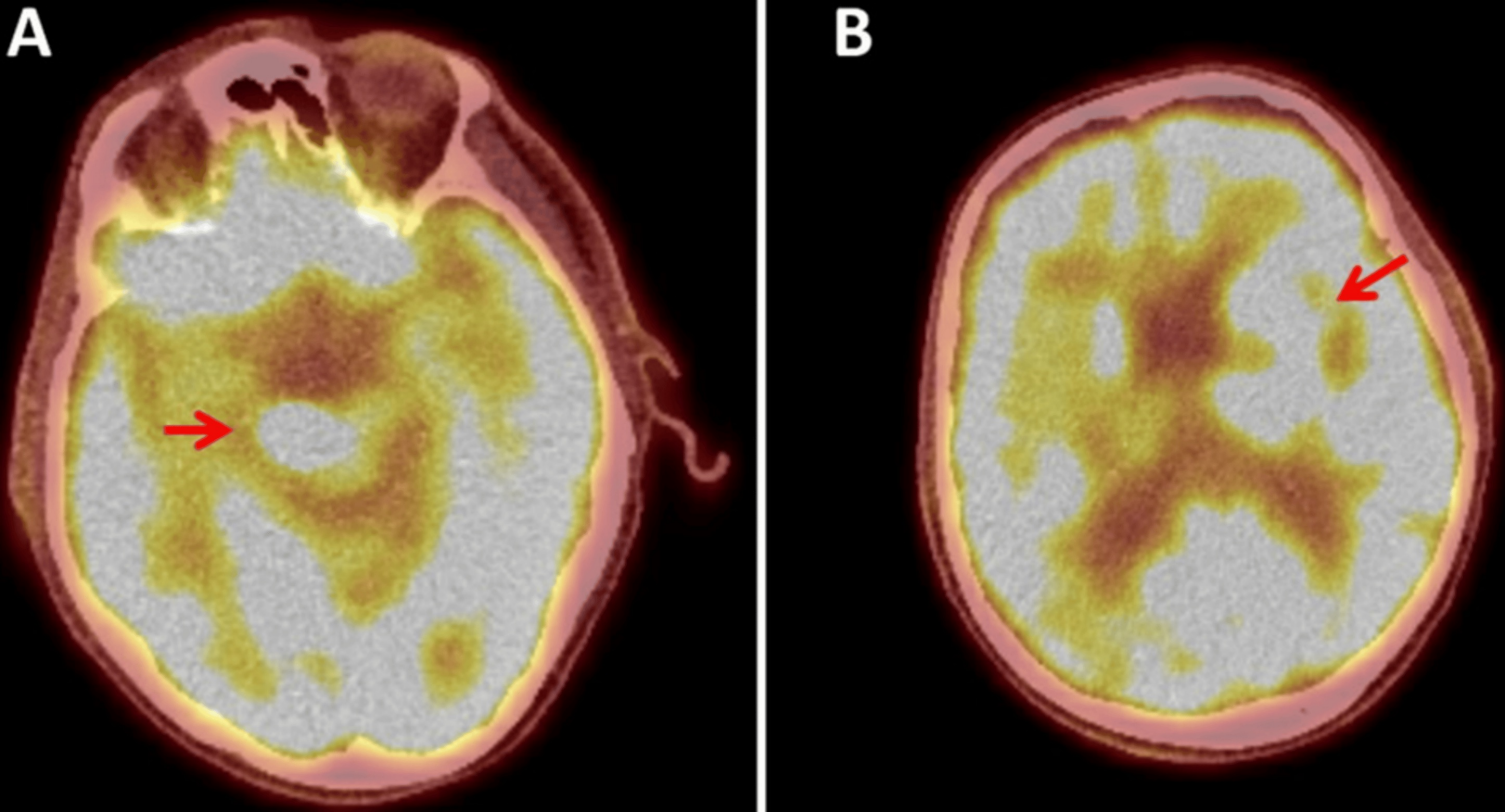
FDG-PET showing hypermetabolism in the right pedunculus cerebri (A) and the left basal ganglia (B). FDG-PET: fludeoxyglucose-18 positron emission tomography

At the age of 86, the patient was readmitted due to progressive gait disturbance and recurrent falls. The clinical neurological examination revealed the mild diffuse weakness of the right upper extremity (M5-), extensive tetra-ataxia with left-sided dominance, reduced Achilles tendon reflexes, and pronounced stance ataxia with a tendency to fall. MRI of the brain showed new onset and size progression in the cerebral pedunculus bilaterally and the left basal ganglia compared to an MRI one year earlier (Figures 2A-2F). CSF examination was normal, without pleocytosis or malignant lymphocytes. In agreement with the MRI of the brain, the PET-CT revealed a hypermetabolic lesion in the left basal ganglia and left cerebral crus (Deauville 5) (Figures 3A, 3B). Clarification of the suspected polyneuropathy was inconclusive.

The patient was treated with dexamethason and underwent seven cerebral radiotherapies without chemotherapy. His current medication on discharge included dexamethasone (4mg/d), entecavir, valacyclovir, amphotericin-B, candesartan, tramadol, trazodone, and pantoprazole.

## Discussion

The patient is interested in PCNSL with an isolated ocular recurrence, which manifested two years later as vitritis on the right and left, and an isolated CNS recurrence another two years later. Despite resection of the PCNSL in the cerebellum and consecutive chemotherapy with RTX, MTX, cytarabine, and procarbacin, the PCNSL relapsed two years later in the form of an isolated vitreoretinal lymphoma. Despite bilateral vitrectomy, the patient suffered a second isolated relapse in the CNS two years later at the age of 86. As the patient was closely monitored after the initial diagnosis and first chemotherapy, it was considered unlikely that the original PCNSL continuously overlapped with the vitreoretinal lymphoma. Another argument against a continuous transition is that the administration of chemotherapy was effective for two years without relapse. A relapse in the form of bilateral vitritis is therefore more likely than a continuum between PCNSL and vitreoretinal lymphoma.

Although PCNSL has been reported in association with secondary ocular involvement [10], the index patient is unique. The ocular lymphoma developed not earlier than two years after the diagnosis of PCNSL because the vitreoretinal lymphoma occurred without CNS involvement, and because CSF examination revealed no pleocytosis respectively meningeosis. The absence of pleocytosis in PCNSL is not uncommon and has been reported occasionally [11]. In a study of 205 patients with PCNSL, only 33 had meningeal dissemination [11]. The likelihood of detecting meningeal dissemination was higher in patients with pleocytosis than in patients without pleocytosis [11]. In a study of 116 patients with PCNSL, pleocytosis was detected in 36% of them [12]. Pleocytosis correlated with positive cytology, whereas CSF protein did not [12]. It should also be borne in mind that primary vitreoretinal lymphoma may be complicated by secondary CNS involvement [13,14] and that in these cases it is difficult to assess at which site the lymphoma first appeared when both occur simultaneously.

The treatment of PCNSL relies on the administration of steroids (e.g., glucocorticoids), chemotherapeutics (e.g., MTX, cytarabine, thiotepa, temozolamide, thalidomide analogs (e.g., lenalidomide, pomalidomide), procarbazine, temozolomib, etoposide, vincristine [15,16]), targeted therapy (e.g., monoclonal antibodies (RTX, nivolumab, immune checkpoint inhibitors (e.g., pembrolizumab)), tyrosine-kinase inhibitors (ibrutinib, orelabrutinib)), immunotherapy (CAR T-cell therapy), ASCT together with high dose chemotherapy, and radiotherapy. Particularly in cases in which PCNSL becomes refractory to an established therapy, monotherapy with ibrutinib can be attempted [17]. Refractory cases are also treated with autologous stem cell transplantation (ASCT). Another newly introduced option for refractory cases is anti-CD19 CAR T-cell therapy with axicabtagene ciloleucel (axi-cel) or tisagenlecleucel (tisa-cel). However, possible side effects of CAR T-cell therapy include cytokine release syndrome (CRS) and immune effector cell‐associated neurotoxicity syndrome (ICANS).

The cause of the polyneuropathy in the index patient is unclear, but it can be assumed that it is due to the neurotoxicity of the chemotherapy. Classical risk factors for polyneuropathy could not be identified in the index patient. However, cytarabine is known to cause neuropathy of the peripheral nerves in rare cases [18,19]. In rare cases, neuropathy has also been reported as a side effect of procarbacin [20].

## Conclusions

In conclusion, this case shows that PCNSL can relapse in isolated secondary ophthalmic lymphoma and in isolated PCNSL a second time. Although glucocorticoids, chemotherapy, and radiotherapy can be very effective, the long-term outcome of patients with PCNSL and vitreoretinal involvement is rather poor. Patients with PCNSL should be closely monitored for extracerebral relapses, including ocular.
